# New Insights into the Role of Metals in Host–Pathogen Interactions

**DOI:** 10.3390/ijms23126483

**Published:** 2022-06-10

**Authors:** Serena Ammendola, Andrea Battistoni

**Affiliations:** Department of Biology, University of Rome “Tor Vergata”, Via della Ricerca Scientifica, 00133 Rome, Italy

Almost eighty years have passed since the publication of the studies by Arthur Schade and Leona Caroline, which we can consider as the first investigations that began to disclose the importance of metals in host–pathogen interactions. In the first of these seminal work, published in *Science* in 1944, these researchers demonstrated that the ability of raw egg white to inhibit the growth of various microorganisms could be overcome by the addition of iron, implying that egg white includes components able to sequester iron and to make it inaccessible to bacteria and fungi [1]. Two years later, the same authors demonstrated that human blood plasma contains a protein component capable of sequestering iron and inhibiting the growth of *Shigella dysenteriae* [2]. The discovery of serum transferrin represented a tremendous advancement in the understanding of iron metabolism in vertebrates and, more generally, of metals in living systems. After these initial studies, for many decades, the investigations on the role of metals in microbial pathogenicity have been focused almost exclusively on the role of iron, somehow following a similar trend in the biochemical research on the structure of proteins, which has long been oriented towards the characterization of abundant and easy-to-purify iron-containing proteins, such as hemoglobin and myoglobin. In relatively recent years, it has been possible to understand that, in addition to iron, other elements of the fourth period of the periodic table such as zinc, iron, manganese, copper, cobalt, and nickel also play important roles in the interaction between microbes and their hosts [3]. Nowadays, we know that the innate immune response to microbial infections encompasses a set of diversified strategies based on the manipulation of the availability of these metals, which include both their sequestration and the possibility of poisoning microorganisms with toxic concentrations of these elements. These host defense mechanisms and other interspecific processes of competition for these nutrients represent important evolutionary forces that led to the development of complex and diversified strategies through which pathogenic microorganisms obtain metals in environments poor in these elements or resist their toxicity. The pathways of use of metals have such a central role in microbial metabolism that indeed represent interesting targets for the developing new antimicrobial strategies, which explains the increasing interest in their elucidation. 

In the context of the urgent need to find new antimicrobial compounds that can help overcome the spread of pan-drug bacterial resistance, some of the studies included in this Special Issue address the possible use of chelating agents or strategies to interfere with iron homeostasis. The contribution by Vinuesa and McConnell [4] provides an overview of the host strategies to maintain low free iron concentration, a description of the main mechanism employed by pathogens for iron acquisition, and a detailed survey of the most relevant studies performed on the four classes of iron chelators in recent decades. The survey includes a comprehensive comparison of the in vitro antimicrobial activity of a range of iron chelators on some of the most challenging resistant bacterial pathogens. It highlights some limitations in their use, such as the ability of pathogens to exploit some of these compounds to their advantage, the low selectivity of some chelators, and the low absorption of synthetic compounds by pathogens. In this context, the authors stress the importance of recent findings reporting an enhancement in the efficacy of some chelators through the addition of chemical moieties that increase the affinity for metals. The second part of the review focuses on the therapeutic properties of Gallium as an iron-mimicry metal that lacks redox activity under physiological conditions. The authors provide a comprehensive evaluation of literature studies on the efficacy of Ga (III) in inhibiting planktonic and biofilm growth of some pathogens. Finally, the promising use of complexes between Ga (III) and chelating compounds such as protoporphyrin IX or deferoxamine is discussed. The authors underline that it will be essential to extend the experimentation of these compounds on clinical isolates, besides elucidating the mechanism of action of these complexes and evaluating the potential toxicity of chelators in patients.

The contribution by Cahill et al. [5] provides an in vitro proof of concept that iron chelators can enhance the activity of antimicrobial compounds. In an attempt to find new approaches to overcome long-term treatments of tuberculosis patients with antibiotics that can cause harmful side effects and can ultimately increase the incidence of resistant strains, the iron chelator desferroxiamine (DFX) was tested in combination with different antimicrobials on *Bacillus Calmette-Guérin* (BCG)-infected macrophages. The bacteriostatic or bactericidal effect of a panel of second-line antimicrobials was tested against BCG in human macrophages to choose the best candidate compounds and to determine their sub-optimal concentration for the combination study. Among them, bedaquiline in combination with DFX showed a better antibacterial effect than bedaquiline alone and concomitantly demonstrated significant immunostimulatory potential revealed by an increase in the secretion of cytokines such as IFN-γ, IL-6, and IL1b.

Remaining in the field of chelators, the study by Ammendola et al. [6] explores the possibility that a specific compound can have different effects on distinct microorganisms. The study shows that *Salmonella* Typhimurium and *Pseudomonas aeruginosa* display significantly different sensitivity toward deferiprone (DFP), an iron chelator successfully used in the treatment of thalassemic patients with Fe overload. DFP was shown to penetrate *P. aeruginosa*, suggesting the presence of an outer membrane protein that can favor the entry of the compound. At the same time, in the case of *S. typhimurium*, DFP exerts effects related to its ability to chelate extracellular iron in competition with bacterial siderophores. The possibility to increase the ability of DFP to cross membranes was explored by testing an array of newly synthesized DFP-derivatives with different lipophilic tails. The study successfully identified a compound that was shown to be more effective than DFP on *S. typhimurium*, while having little effect on *P. aeruginosa*, because of the different mechanism of action of DFP on the two microorganisms. On the one hand, the results presented in this paper support the idea that the chemical modification of an already known chelator is a promising strategy for enhancing its antimicrobial activity. On the other hand, they suggest that the choice of a metal chelator should be carefully evaluated also considering the target pathogen. 

The contribution by Lopez-Berges et al. [7] concerns the importance of iron homeostasis in the virulence of eukaryotic pathogens. The filamentous fungus *Aspergillus. fumigatus* is one of the most common airborne fungal pathogens in humans, causing opportunistic infections that can be life-threatening in immunocompromised patients. *A. fumigatus* regulates iron uptake in response to its availability mainly through the bZIP factor HpaX, in which the gene is highly transcribed in iron-limiting conditions and repressed by the metal. The authors showed that, besides its transcriptional regulation, the HpaX factor is strictly regulated post-translationally. They showed that, in the switch from iron-limiting to iron-replete conditions, HpaX is degraded within a few minutes. The analysis of the interactome of HpaX in different iron-availability conditions shows that its degradation is a consequence of modifications such as ubiquitination and sumoylation and can be reduced in Fbx22 or SumO mutant as well as by specific point mutations in HpaX. Interestingly the authors show that a quick degradation of HpaX is necessary for a correct response to iron, finding that *A. fumigatus* mutants with an impaired ability to post-translationally modify this regulator have an evident growth defect related to iron exposure.

Two other contributions to this Special Issue concern iron acquisition via siderophores in *P. aeruginosa*. The paper by Philem et al. [8] investigated the role of PvdF, a hydroxyornithine transformylase enzyme, in the synthesis of pyoverdine, the siderophore with the highest affinity for iron among those synthesized by this opportunistic pathogen. Through a mass spectrometry assay, their study confirmed the predicted activity of the enzyme. At the same time, site-directed mutagenesis experiments investigated the role of some amino acid residues predicted to be required for enzymatic activity. Interestingly, their study reveals that some mutations that are compatible with the maintenance of the enzyme activity in vitro, strongly reduce its activity in vivo. This observation could be related to the demonstration that PvdF forms a stable protein–protein complex with PvdA, the enzyme that generates L-N5 hydroxyornithine, the precursor used by PvdF. This finding implies that PvdF is a component of the siderosome, i.e., the multienzyme complex mediating the synthesis of pyoverdine, and suggests that the activity of the enzyme could be modulated by the interaction with the other components of the complex.

The study by Gasser et al. [9] addresses the interesting problem of the competition for iron among different microorganisms. It is known that *P. aeruginosa*, in addition to being capable of synthesizing the siderophores pyochelin and pyoverdine, can also acquire iron by internalizing the siderophores produced by other bacteria. For example, it can pirate enterobactin (ENT), the siderophore produced by *Escherichia coli. P. aeruginosa* can utilize the iron bound to ENT through its hydrolysis carried out by the periplasmic esterase PfeE. Through co-cultivation experiments in an iron-deficient medium, this study revealed that the absence of PfeE renders *P. aeruginosa* unable to use iron complexed to ENT. Even in the absence of PfeE *P. aeruginosa* can repress the growth of *E. coli* through the production of its own siderophores. Moreover, a *P. aeruginosa* strain unable to produce endogenous siderophores is still capable of growing in equilibrium with *E. coli* if it can utilize the iron bound to ENT. However, this mutant strain is outcompeted by *E. coli* if the production of PfeE is abolished. Overall, these data reveal that PfeE plays a central role in *P. aeruginosa* ability to thrive in bacterial communities containing bacteria that produce ENT.

Two other contributions by the group of Magdalena Rowińska-Żyrek investigated the metal-binding properties of peptides with potentially interesting biological activities. The paper by Miller et al. [10] focuses on surfactant-associated anionic peptides initially isolated from the ovine pulmonary surfactant. These peptides have a strong antibacterial activity, enhanced by zinc, against the sheep pathogen *Mannheimia haemolytica*. Their study focused on the ability of these peptides to bind both zinc and copper. The most surprising aspect of their study is that these peptides are highly species-specific and have no effect on a panel of microbial pathogens including Gram-negative and Gram-positive bacteria and fungi. Furthermore, the binding of the metals does not seem to enhance the membrane-disrupting ability of the peptides. This suggests that these peptides selectively target a protein on the surface of *M. haemolytica* and that zinc binding favors the acquisition of an optimal peptide conformation for this interaction. 

Finally, the paper by Witkowska et al. [11] analyzed the nickel-binding properties of N-terminal fragments and histidine-rich fragments of Hpn-like protein from two *Helicobacter pylori* strains. This study may help to understand the role of this protein in *H. pylori* nickel metabolism.

Taken together, the various contributions to this thematic issue highlight different aspects of the importance of metals for pathogenic microorganisms and underline how, almost eighty years after the studies of Schade and Caroline, there are still many aspects to clarify to deeply understand the role of metals in host–pathogen interactions. However, what is already very clear today is that metal homeostasis is of such central importance in bacterial physiology that it represents a promising target for novel antimicrobial approaches. New drugs capable of interfering with the ability of pathogens to acquire essential elements and/or to eliminate toxic metals could become formidable tools in countering the growing problems caused by the spread of resistance to current antibiotics.

## Data Availability

Not applicable.
